# An Unusual Presentation of COVID-19: Hemorrhagic Pericardial Effusion With Tamponade Physiology

**DOI:** 10.7759/cureus.13438

**Published:** 2021-02-19

**Authors:** Brian Foster, Adnan Liaqat, Anjula Chib, Scott S Bolton, Arthur C Kendig

**Affiliations:** 1 Internal Medicine, Southeast Health Medical Center, Dothan, USA; 2 Radiology, Southeast Health Medical Center, Dothan, USA; 3 Cardiovascular Disease, Southeast Health Medical Center, Dothan, USA

**Keywords:** hemorrhagic pericardial effusion, cardiac tamponade, pericardial effusion, viral pericarditis, covid pericarditis, hemorrhagic pericarditis, mri cardiac, covid-19, covid and heart, covid

## Abstract

A 44-year-old woman with a history of factor V Leiden deficiency and recurrent pulmonary emboli was diagnosed with coronavirus disease 2019 (COVID-19) three weeks prior presented to her local ED with severe chest pain. She was found to have a large hemorrhagic pericardial effusion by cardiac MRI with echocardiographic signs of tamponade. She underwent the creation of a pericardial window and was treated with colchicine with improvement in symptoms.

## Introduction

Given the overwhelming amount of information being published surrounding the various clinical manifestations seen in patients suffering from severe acute respiratory syndrome coronavirus 2 (SARS-CoV-2) virus and coronavirus disease 2019 (COVID-19), few case reports exist defining the pericardial manifestations. Here, we present a case describing a middle-aged female that was found to have a hemorrhagic pericardial effusion, thought to be a late sequela of her previously asymptomatic COVID-19 infection.

## Case presentation

**Figure 1:**
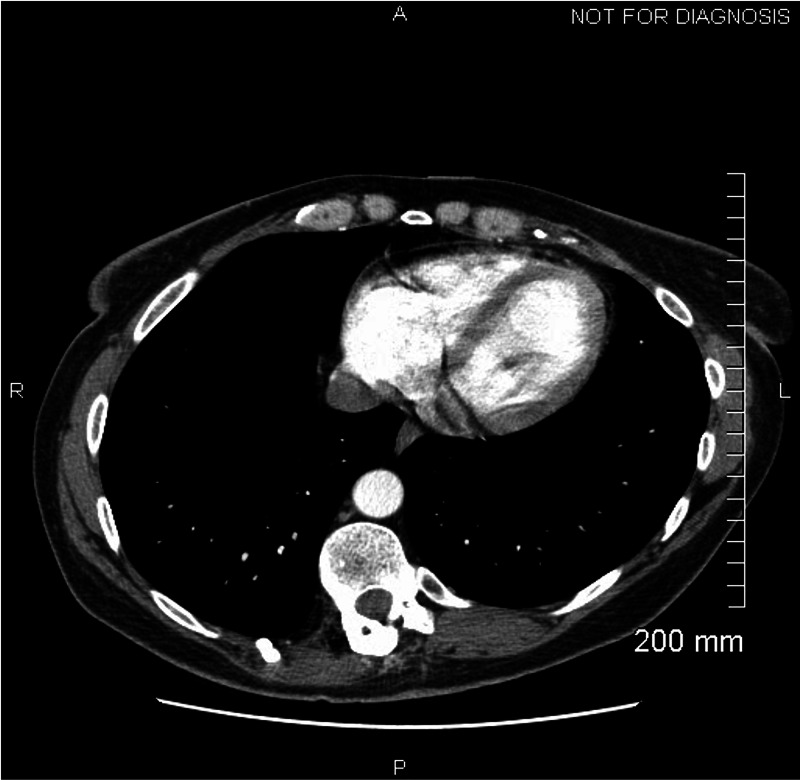
CT chest with contrast at a local hospital prior to transfer demonstrating no effusion.

**Figure 2:**
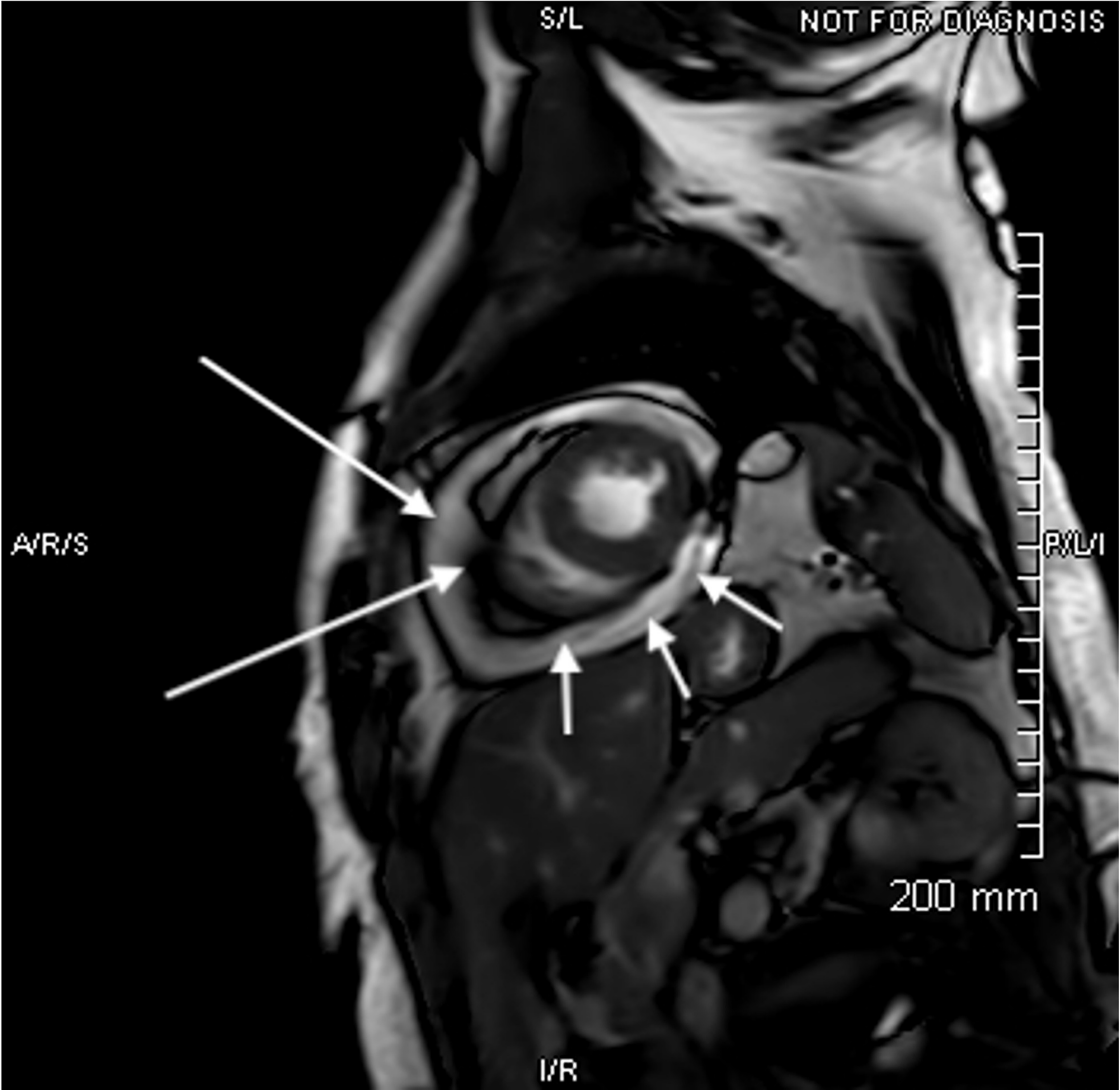
Cardiac MRI. Short axis steady-state free precession image demonstrating large pericardial effusion (short arrows) with blooming along the visceral and parietal pericardium (long arrows) due to hemosiderin deposition in the setting of a hemorrhagic effusion.

A 44-year-old Caucasian female nurse with a history of factor V Leiden deficiency, recurrent pulmonary emboli on chronic warfarin therapy, and hypothyroidism presented to her local emergency department with severe, substernal, and positional chest pain (worse when supine) that radiated to her left shoulder and jaw. She reported COVID-19 positive testing three weeks prior to the presentation including a two-week self-isolation period after her diagnosis. She denied a history of shortness of breath, fever, myalgias, GI complaints, or any other concerning symptoms for COVID-19 infection. Thus, she assumed her result was a false positive. Her local electrocardiogram (EKG) was negative for acute ST-T changes and a CT angiogram was negative for pulmonary emboli or pericardial effusion (Figure 1). She was transferred to our facility for further evaluation of non-ST elevation myocardial infarction (NSTEMI) after a mild troponin elevation was noted (0.4 ng/mL). On admission, her EKG revealed borderline (but not diagnostic) diffuse ST elevation, PR interval depression in leads II, III, aVF, and mild elevation of the PR interval in aVR. A chest x-ray was negative for any acute cardiopulmonary process. Pertinent laboratories included a white blood cell count of 11.9 x10^-3^/uL (ref 4.5-10 x10^-3^/uL), erythrocyte sedimentation rate (ESR) of 10 mm/hr (ref 0-30 mm/hr), and a C-reactive protein was found to be 0.75 mg/dL (ref 0-0.99 mg/dL). The international normalized ratio (INR) at the time of admission was mildly supratherapeutic at 3.16 (goal INR 2-3). Her repeat troponin after transfer was found to be 0.4 ng/mL (ref 0-0.03 ng/mL). Repeat COVID-19 testing was performed and was positive. A D-dimer was found to be elevated at 273 (ref 0-230 ng/mL) and LDH elevated to 476 (91-200 IU/L). Cardiac MRI revealed acute hemorrhagic pericardial effusion with probable pericardial tamponade (Figure 2). An echocardiogram demonstrated a large pericardial effusion with right ventricular (RV) diastolic invagination consistent with tamponade.

## Discussion

There are few cases in the literature describing cardiac manifestations of COVID-19 and fewer isolated cases such as this describing the development of pericarditis and pericardial effusions in COVID-19 infected patients. At this point, there are no reported case reports describing patients presenting with chest pain as the sole complaint this far removed from initial positive testing [1-5]. Given our patient had a positive COVID-19 test three weeks prior to her presentation (with a two-week isolation period), it is a reasonable conclusion that her hemorrhagic pericardial effusion is related to COVID-19, and thus this is a previously unknown late manifestation of COVID-19 infection. There is an uncertainty that her COVID-19 infection completely explains the hemorrhagic pericardial effusion given her elevated INR on admission. However, a mildly supratherapeutic INR of 3.16 alone resulting in spontaneous hemorrhagic pericarditis would be unlikely. It is more likely that in the setting of COVID-19 pericarditis, anticoagulation may have potentiated the hemorrhagic response. Although the pathophysiology is not completely understood, current literature attributes the development of hemorrhagic pericarditis in COVID-19 patients to the systemic inflammatory response and subsequent cytotoxic and immune-mediated effects related to SARS-COV-2 [3]. Widely available validated testing to assess for COVID-19 infection in pericardial fluid does not exist, thus the fluid from our patient was not able to be tested for confirmation of the presumptive diagnosis [3]. The development of further testing for the detection of COVID-19 in a pericardial fluid is necessary. In terms of management, there are currently no published guidelines for the management of hemorrhagic pericarditis secondary to presumed COVID-19 infection. In the few other reported cases in the literature, patients were treated with colchicine, hydroxychloroquine, steroids, and antivirals. As stated above, we started our patient on colchicine and saw a fairly rapid positive clinical response, with improvement in her symptoms within less than 24 hours. Classically, nonsteroidal anti-inflammatory drugs (NSAIDs) plus colchicine or corticosteroids plus colchicine are used to treat pericarditis [6]. However, given her supratherapeutic INR, NSAIDs were contraindicated. Future studies are needed to determine a standard treatment regimen and the pros/cons of various treatment options.

## Conclusions

As COVID-19 positive patients are evaluated, it is important to continually consider the extrapulmonary manifestations of this infection, even in the absence of additional symptoms. Hemorrhagic pericardial effusion with subsequent cardiac tamponade must be considered as a potentially lethal late manifestation of COVID-19 infection. To our knowledge, this is only the second reported case of hemorrhagic pericardial effusion with tamponade physiology secondary to COVID-19 infection reported.
